# Polypus of the Uterus, Treated by the Internal Administration of Ergot

**Published:** 1875-05

**Authors:** Daniel F. Collins

**Affiliations:** New York


### POLYPUS OF THE UTERUS, TREATED BY THE INTERNAL ADMINISTRATION OF ERGOT.

BY DANIEL F. COLLINS, M. D., NEW YORK.

Mrs. E. S., a short, thin woman of sallow complexion, and the mother of four children, sent for me to attend her, as she said, for a "womb trouble." On calling, I found her exhausted from uterine hemorrhage and in a very dangerous condition.

In answer to my questions, she stated that she was sick and in delicate health for the past six months, and had suffered a great deal from "flooding," and that these attacks generally came on every eight or ten days. But for the last two months she lost more or less blood all the time.

Having checked the hemorrhage I left, promising to call the following day.

On calling next morning I found her free from any symptom of flooding, but in a very weak condition. On introducing my finger through the os uteri, I found at the upper posterior portion of the organ a round substance or tumor about the size of a small orange; passing my finger around it I found it was impossible to pass even the point of my finger between the base of the tumor and the side of the womb, and that the tumor seemed to be closely attached to the wall of the uterus. The patient being very weak and nervous from loss of blood, I deferred further examination until next morning, which further examination satisfied my mind that it was impossible to remove the tumor *in its present* condition and relation to the uterine wall without a considerable and dangerous loss of blood, which, considering the weak and exhausted condition of my patient, I did not feel justified in risking.

After a consideration of the case, I decided on giving moderate doses of ergot in combination with a little opium, in order to bring on such contractions of the uterus as would separate the polypus or tumor from the uterine wall, sufficient for me to either strangulate the tumor or remove by excision.

On paying my visit next morning, the patient complained of "bearing down pains," and said that she suffered as much as if she were in the beginning of labor.

On making an examination I at first found considerable difficulty in introducing my finger through the os, owing in the first place to the state of contraction the uterus was in, and secondly to the tumor pressing down from the fundus of the womb, and as if blocking up the passage.

On succeeding in introducing my finger, I found that the body of the tumor or polypus had entirely separated from the wall of the uterus, and was but now held by a small pedicle about three quarters of an inch in length, by a little less than half an inch in diameter.

After a careful examination I could find no trace of pulsation in the pedicle, but found it soft, and to the touch not unlike that of the umbilical cord.

Taking a gentle but firm hold of the polypus, I turned it round and round several times and then withdrew my hand.

After cautioning my patient against any unnecessary exertion, and telling her to send for me if there was any change in her condition, I left.

On the following morning I found on examination that the pedicle had softened a good deal owing to the twisting the previous day, and discovering no trace of pulsation in it I at once passed up a curved blunt-pointed scissors, and with one clip severed the connection between the polypus and the wall of the uterus. I immediatly gave the patient a dose of ergot which brought on firm contractions in a short time. The polypus was expelled, the patient not having lost a teaspoonful of blood.

The polypus was of fibroid character and measured two and one half inches long by two and one quarter inches in diameter, and was hollow, containing a lot of grumous blood.

The patient rapidly recovered and is now strong and healthy, has had no hemorrhage since the removal of the polypus, and is quite free from "womb trouble."

Having examined several of the latest works on uterine diseases, I cannot find in any of them any allusion to the exhibition of ergot in cases similar to the above. That is, giving ergot as a means for separating, as far as its attachments will allow, the body of a tumor or polypus from the wall of the womb before removal.

Churchill, in his treatise on the diseases of women, says in speaking of the treatment of "polypus of the uterus:" "In order to hasten the expulsion of the polypus through the os uteri, it will be advisable to give ergot; and more especially as, even if there be no polypus, its effects in restraining the hemorrhage will be beneficial. If the polypus appear and disappear we may employ ergot, and at its reappearance fix it with Museux forceps and draw it down and tie it." In the above quotation, ergot is advised to be used simply to hasten the expulsion of the tumor, the forceps being recommended as a means of drawing the tumor down.

McClintoch, in his work on the diseases of women, makes no mention of ergot as a means of lengthening the neck of a polypus before removal, or for separating the tumor as far as possible from the uterine wall.

Another question that presents itself in considering the above case is: In many of the cases that are treated as fibroid tumors of the uterus, in which the physician finds the tumor firmly and closely attached to the wall of the uterus, and almost imbedded in it; would not the careful use of ergot in many of these cases, change our diagnosis from that of fibroid tumor to simple polypus, and consequently alter our treatment in many cases, from giving temporary relief and using palliative measures, to a permanent cure by the removal of the morbid growth?

That the judicious use of ergot itself, or in combination with opium, will, in a large number of cases, materially help and simplify the operation of removal, I have little doubt. It will do so by producing sufficient artificial contraction of the uterus to enable that organ to separate, as far as its attachments will permit, the morbid growth from its walls. In doing so, the danger of including a portion of the wall of the uterus is removed, or at least greatly lessened, and in many cases a dangerous hemorrhage avoided.—*New York Medical Record.*

